# Sexual and reproductive health behaviors of female sex workers in Dhaka, Bangladesh

**DOI:** 10.1371/journal.pone.0174540

**Published:** 2017-04-03

**Authors:** Tasnuva Wahed, Anadil Alam, Salima Sultana, Nazmul Alam, Ratana Somrongthong

**Affiliations:** 1 College of Public Health Sciences, Chulalongkorn University, Bangkok, Thailand; 2 Research to policy Limited, Mirpur, Dhaka, Bangladesh; 3 Maternal and Child Health Division, icddr,b, Dhaka, Bangladesh; 4 HIV/AIDS Sector, Save the Children, Gulshan, Dhaka, Bangladesh; 5 University of Montreal Hospital Research Center (CRCHUM), Montreal, Quebec, Canada; National Institute of Health, ITALY

## Abstract

**Objectives:**

The objective of this study was to document sexual and reproductive health (SRH) practices among female sex workers (FSWs) including abortion, pregnancy, use of maternal healthcare services and sexually transmitted infections (STIs) with the aim of developing recommendations for action.

**Methods:**

A total of 731 FSWs aged between 15 and 49 years were surveyed using a stratified sampling in Dhaka, Bangladesh. A workshop with 23 participants consisted of policy makers, researchers, program implementers was conducted to formulate recommendations.

**Results:**

About 61.3% of 731 FSWs reported SRH-related experiences in the past one year, including abortion (15.5%), ongoing pregnancy (9.0%), childbirth (8.3%) or any symptoms of STIs (41.6%). Among FSWs who had an abortion (n = 113), the most common methods included menstrual regulation through manual vacuum aspiration (47.8%), followed by Dilation and Curettage procedure (31%) and oral medicine from pharmacies (35.4%). About 57.5% of 113 cases reported post abortion complications. Among FSWs with delivery in the past year (n = 61), 27.7% attended the recommended four or more antenatal care visits and more than half did not have any postnatal visit. Adopting sustainable and effective strategies to provide accessible and adequate SRH services for FSWs was prioritized by workshop participants.

**Conclusion:**

There was substantial unmet need for SRH care among FSWs in urban areas in Dhaka, Bangladesh. Therefore, it is important to integrate SRH services for FSWs in the formal healthcare system or integration of abortion and maternal healthcare services within existing HIV prevention services.

## Introduction

Female sex workers (FSWs) are at high risk of mortality and morbidity related to both sexual and reproductive health (SRH), particularly from unsafe abortion and pregnancy-related complications including sexually transmitted infections (STIs). There is an increasing trend in the FSW population in many Asian countries, as women are often entering the sex trade at younger ages than in the past [1]. In Bangladesh, a small country in Asia, approximately 0.22% of 15–49 aged female population is engaged as FSWs [2]. Commercial sex is not legal in Bangladesh, and as a result, there are limited services available in formal healthcare systems which are an important public health issue.

FSWs are at high risk of becoming pregnant because of their reproductive age, poor negotiation power for condom use, and the high number of clients they entertain. The prevalence of consistently using condoms with new clients in the past week among brothel-based, hotel-based and street-based FSWs in Dhaka was 28.4%, 7.8% and 43.3% respectively [3]. The number of clients entertained by each FSW in Bangladesh is one of the highest in Asia. According to the 7^th^ round behavioral surveillance survey, brothel-based FSWs entertained an average of 20 clients per week [4]. A study showed that 61% of hotel-based FSWs had become pregnant once or more and 51.4% reported using menstrual regulation (MR) procedure to terminate the pregnancy [5]. However, some FSWs continue their pregnancy up to 28 weeks of gestation or more. One study demonstrated that 12% of brothel-based FSWs had a child less than one year of age [6]. Thus, it is necessary to understand their behaviors for seeking pregnancy and delivery care.

Bangladesh is committed to reduce maternal mortality and morbidities through various interventions and research which have been implemented targeting rural, urban slum and non-slum areas. The Government of Bangladesh also established a supportive network of abortion clinics which carried out more than one million pregnancy terminations by MR or abortion [7].The Bangladesh Demographic and Health Survey (BDHS) 2015 demonstrated that in urban areas, women are more likely to seek antenatal care from medical personnel compared with rural areas (74% and 52% respectively) [8]. However, women in urban slum areas are less likely to seek four antenatal visits (28.5% vs. 58%) or give birth with a skilled attendant (37% vs68%) than women in urban non-slum areas [9]. Moreover, FSWs are particularly at risk for STIs [10] and STIs have been associated with a number of adverse pregnancy outcomes including spontaneous abortion, stillbirth, prematurity, low birth weight (LBW), postpartum endometritis, and various sequelae in surviving neonates [11]. There is evidence that FSWs face stigma and discrimination in seeking health services [12]. However, limited information is available on abortion, pregnancy and delivery behaviors and/or care-seeking for STIs among FSWs, even though this group of women is particularly vulnerable due to limited financial affordability, illiteracy, discrimination, social exclusion and segregation [13].

Under the stewardship of the National AIDS/STD Program (NASP) of Bangladesh, Save the Children implements programs to support access to HIV prevention services for FSWs through the Rolling Continuation Channel (RCC) grant. There are two packages of interventions that are being implemented to reach approximately 28,600 FSWs in 51 districts through 100 Drop in Centers (DICs) and 10 outreach offices. DICs are located in high priority areas, with the largest numbers of un-served FSWs, higher prevalence of HIV and STI among FSW, geographical vulnerability and field-level experience. DICs provide a variety of services including behavior change education, free condoms, STI & general health service and effective referral services for voluntary counseling and testing (VCT) and maternal & child health (MCH) services [14,15]. The present study was conducted in DIC with the aim of understanding sexual and reproductive health behaviors of FSWs during pregnancy, delivery and symptoms related to STIs to inform the design of essential interventions to improve SRH related health outcomes.

## Methods

### Study design, setting and population

A cross-sectional descriptive study was conducted in 2015 among FSWs in Dhaka city. Dhaka, the capital city of Bangladesh is highly populated with about 15 million people living in 315 mi^2^ areas with an estimated population growth of 4.2% per year [16]. The study was conducted in the DIC coverage areas in Dhaka, which were implemented by Save the Children with support from the Global Fund. The study population was comprised of residence, hotel, and street-based FSWs who were working in Dhaka city during the study period.

### Sample size

The study sample size was estimated at 720, based on standard parameters, such as-proportion of FSWs having SRH related experiences (eg., 50% in this case as proportion was unknown), 95% confidence interval,5% precision of errors, design effect of 1.5 and 20% non-response rate.

### Sampling

A total of 25 DICs were included in the administrative data supplied by Save the Children in Bangladesh. Three DICs were randomly selected using a stratified sampling technique for the quantitative component of the study. At first, all 25 DICs were divided into three strata based on numbers of FSWs. A DIC was considered large (DIC with ≥300 FSWs), medium (DIC with 200–299 FSWs) and small (DIC with <200 FSWs).One DIC from each strata was randomly drawn and all FSWs of the selected DICs were invited to participate for a quantitative face to face interview.

### Data collection technique

Data for this study were collected in two phases. Phase I included a quantitative survey with 731 FSWs. In Phase II, a workshop was conducted to identify barriers in implementation of SRH related services for FSWs, re-examining the findings from Phase I to formulate a policy brief and program recommendations.

#### Phase 1 Survey

A survey was administered with 731 FSWs from selected DICs. The data collection team included four data collectors and one field supervisor. The team was trained on the survey questionnaire which had been shown in **S1 Table**.

A literature review was undertaken by the principal investigator to formulate the questionnaire which contained questions on socio-demographic characteristics and SRH issues, such as- abortion, maternal healthcare and STIs. Assessment of each question was carried out by a medical doctor with expertise on sexual and reproductive health to ensure content validity. Moreover, the questionnaire was reviewed by experts in the field of sexual and reproductive health at icddr,b, Save the Children in Bangladesh and Chulalongkorn University. Three SRH-related research specialists were invited to give an overall score of ‘-1’ (further revision is required) or ‘0’ or ‘+1’ (the questionnaire is okay and can be used for data collection) on the questionnaire. We got ‘+1’ score on the questionnaire from each of them. This questionnaire was also field-tested with FSWs in another DIC that was not selected for survey data collection.

DIC staff developed a list of FSWs in their catchment area, including identifying information. The data collectors conducted the interviews at the DICs. They also visited condom distribution locations, where outreach workers distribute condoms to FSWs who were not available at the DIC.

#### Workshop

In Phase 2, a half-day workshop was conducted with 23 participants from the Government of Bangladesh health department, and national and international organizations and partners **(Table 1)**. The participants were key national policy makers, FSW-based health program implementers, health system specialists, public health researchers including scientists from reproductive health and HIV fields. At first, the principal investigator prepared a list of key participants involved in SRH-related interventions from her knowledge and experience. She shared this list with the other investigators of this study and updated the list based on their feedback. All identified participants were invited through email as well as delivered printed invitation letters at their offices. One day before the workshop, participants were reminded via email and telephone. On the day of the workshop, participants from almost all organizations were present and successfully contributed to the workshop. One participant from the office of the Director General Family Planning of Government of Bangladesh was out of town; therefore she could not attend. The reasons for absence for the other five participants who did not attend were unknown. The Principal Investigator presented the findings and recommendations which were obtained from Phase I data collection among the participants. After the presentation, the presenter invited participants to an open discussion for re-examining the recommendations and for giving their opinions to reach better policy implications for FSW. A note taker was appointed to keep notes on the discussion. Audio recorders were also used to record the discussion of participants.

**Table 1:** Number of participants by type of organization.

Name of organization	Type of organization	Number of participants
National AIDS/STD Control and National AIDS/STD Program	Public	1
Save the Children	INGO	2
Institute of Epidemiology, Disease Control and Research (IEDCR)	Public	1
Light House Consortium-FSWI	NGO	2
Bangladesh Women Health Coalition (BWHC)	NGO	1
Durjoy Nari Songha (DNS)	DNS	1
Marie Stopes	NGO	1
James P Grant School of Public Health (JPGSPH)	BRAC University	1
Young Power in Social Action (YPSA)	NGO	1
Research, Training and Management (RTM) International	NGO	1
Social Marketing Company (SMC)	NGO	1
icddr,b	International research organization	10
	Total	23

### Data analysis

#### Phase 1 Survey data

ASP.Net and SQL server were utilized for data entry. Data editing and analysis were done using SPSS software (version 20). The percent distribution of background characteristics, SRH experiences, and service utilization were computed. Continuous variables were categorized based on frequency distribution. Background characteristics included: age, education, place of residence, sex trade characteristics as well as reproductive health characteristics. SRH experience variables included currently pregnant, abortion, delivery or STI in last year. Service utilization variables included: use of antenatal care, delivery care, post-natal care, and reported STI symptoms. Chi-Square test and Fisher’s exact test was calculated to assess differences in SRH experiences across background characteristics.

#### Phase II Workshop data

Workshop data were analyzed using content analysis. A transcript was prepared from raw notes from the note takers and audio recordings. This transcript was assigned to the atlas.ti data management software. The coding was done through continuous reading. Based on the compilation of codes, a summary of discussions and recommendations was developed by investigators, taking into considerations the issues raised in the workshop; for example, sustainability issues and implementation of SRH interventions. Quotes were used to express the voice of participants.

#### Ethics approval

The study was approved by the Research Ethics Review Committee at Chulalongkorn University, Thailand (Protocol No: 178.1/58). A proportion of the FSWs were illiterate and it was suspected that they were not comfortable signing or writing their names in the consent form. Moreover, fear of recognition as FSWs may lead to disagreement for signing the consents. Considering the situation, the Ethic Committee approved the protocol to get verbal consent from adult FSWs. The Committee also approved verbal consents from the guardians of FSWs who were 15 to 17 years old. Once a guardian gave verbal consent, a verbal assent was collected from that under 18 FSW. After describing the aspects of the study, the data collectors asked the respondent about her willingness to participate in a one on one interview If a respondent agreed to participate, data collector put her (data collector) own signature with date on the consent form and preserved.

## Results

### Phase I: Results of survey

#### Socio-demographic characteristics

Almost half of FSWs’ age ranged from 25 to 34 years (46.7%), with more than three-quarters of FSWs’ with 0 to 5 years of schooling (84.6%). About one-third have been performing commercial sex for three to seven years (36.5%), while almost three-quarters conducted a sex act four or more times per day (72.1%). The large majority had a childbirth experience (84.4%) and a half had an abortion experience (52%) in their lifetime. Duration of involvement with sex work was found to be related with all types of SRH experiences (e.g. abortion/pregnancy/delivery) except STIs (p<0.05) (Table 2).

**Table 2:** Socio-demographic characteristics by type of SRH-related experiences (n = 731).

Characteristics	Abortion in last one year (n = 113)%	Currently Pregnant (n = 66)%	Delivery in last one year (n = 61)%	STIs in last one year (n = 304)%	Any SRH experiences (n = 448)%
**a. Socio-demographic characteristics**					
**Age group (in years)**					
15–24	37.2	37.9	39.3	28.6	31.2
25–34	43.4	54.5	47.5	46.4	46.7
35–49	19.5	7.6	13.1	25.0	22.1
p value	ns	*p = 0.002	*p = 0.043	ns	*p = 0.024
**Completed years of schooling**					
0–5 years	84.1	86.4	83.6	84.5	84.6
6 to more years	15.9	13.6	16.4	15.5	15.4
p value	ns	ns	ns	ns	ns
**Place of residence**					
Open space^@^	22.1	25.8	36.1	24.3	25.0
Slum	30.1	28.8	26.2	29.3	28.8
Residential area	46.0	39.4	34.4	45.7	44.6
Rehabilitation centre of NGOs	1.8	6.1	3.3	0.7	1.6
p value	ns	^∞^p = 0.005	^∞^p = 0.031	^∞^ns	^∞^ns
**Monthly income (in BDT^£^)**					
≤10000	43.4	51.5	65.6	53.9	52.7
>10000	56.6	48.5	34.4	46.1	47.3
p value	*p = 0.029	ns	*p = 0.037	ns	ns
**b. Sex trade related characteristics**					
**Duration of involvement in sex work (in years)**					
0–2	32.7	33.3	31.1	29.9	32.0
3–7	48.7	48.5	50.8	37.8	49.6
≥8	18.6	18.2	18.0	32.2	18.4
p value	*p = 0.002	*p = 0.029	*p = 0.023	ns	*p = 0.003
**Number of sex act per day**					
1–3 times	24.8	37.9	26.2	28.3	27.9
4–5 times	35.4	30.3	39.3	37.8	36.6
> = 6 times	39.8	31.8	34.4	33.9	35.5
p value	ns	ns	ns	ns	ns
**c. Reproductive characteristics**					
**Number of childbirth in lifetime (Para)**					
0	16.8	24.2	0	16.1	15.6
1	30.1	30.3	29.5	26.3	28.8
2	30.1	30.3	36.1	27.0	28.1
3 or more	23.0	15.2	34.4	30.6	27.5
p value	ns	*p = 0.054	p = 0.000^∞^	ns	ns
**Number of abortion in lifetime**					
0	0	72.7	68.9	49.0	48.0
1	69.0	19.7	16.4	29.3	33.7
2 or more	31.0	7.6	14.8	21.7	18.3
p value	ns^∞^	*p = 0.006	*p = 0.043	*p = 0.006	*p = 0.000

^@^Open space refers street/Park/Foot over bridge/Stadium/Open space of Theater

^£^1 BDT = 79 US$

*p<0.05 for Pearson chi-square test

^**∞**^p<0.05for Fisher’s exact test, ns = not significant

Note: The significant level was measured among all 731 FSWs by selected SRH experiences.

#### Prevalence of sexual and reproductive health experiences

**Fig 1:**
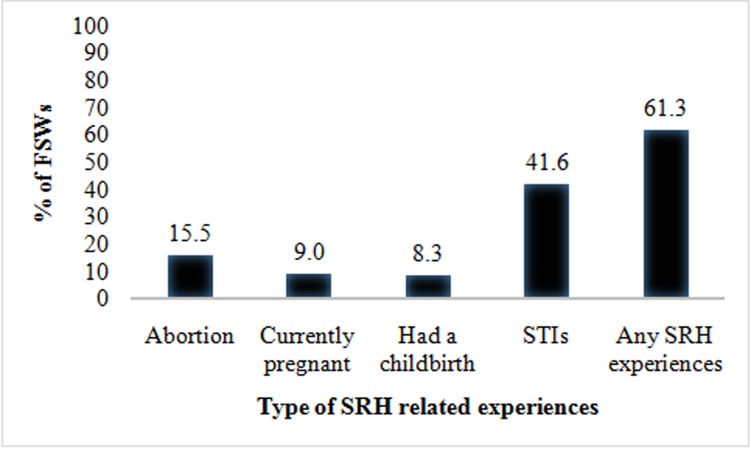
Proportion of FSWs who reported SRH experience in last one year by type of experience (n = 731).

The prevalence of current pregnancy, abortion, childbirth and STI symptoms in the last year were 9%, 15.5%, 8.3% and 41.6% respectively. Overall, 448 of 731 FSWs (61.3%) reported any SRH related experience (eg., abortion or current pregnancy or childbirth experience or STIs) in last one year **(Fig 1)**.

#### Experiences related to abortion

- Management of abortion: Respondents reported MR through manual vacuum aspiration (MVA) (47.8%) and Dilation and Curettage (D & C) (31%) for management of abortion. About one-third of respondents (35.4%) reported using medicine to induce abortion. NGO facilities (15.0%), drug shop/pharmacies (16.8%) and home by unskilled providers (16.8%) were used by almost same proportion of respondents **(Table 3)**.

**Table 3:** Management of abortion among FSWs who reported having an abortion in last one year (n = 113).

Characteristics	%
**Type of management of abortion**^@^	
MR by manual vacuum aspiration (MVA)	47.8
Dilation and Curettage (D & C)	31.0
Allopathic medicine	35.4
Herb/spiritual/eating pineapple	5.3
**Areas of management of abortion**^@^	
Within their locality (In Dhaka city)	74.3
Outside of their locality (In Dhaka city)	18.6
In native village/outside Dhaka city	8.8
**Places/sources of management of abortion**^@^	
Public health facilities	23.9
For profit private health facilities	35.4
Not for profit private (NGO) facilities	15.0
Home by skilled providers	9.7
Drug shop/Pharmacy	16.8
Home by unskilled provider/self/relatives/neighbor	15.9

^@^Multiple responses.

- Management of abortion related complications: Of the 113 FSWs reporting an abortion in the last year, 57.5% reported at least one complication after abortion and about half (49.2%) suffered from excessive bleeding. Lower abdominal pain (40.0%) and severe weakness (43.1%) were also reported by respondents. About one-fourth (24.6%) of FSWs reported blurry vision or symptoms of sexually transmitted infections (STIs) or foul smelling discharge or incomplete abortion. Qualified doctors (35.4%) and paramedic/family welfare visitors (26.2%) were cited as providing care for FSW’s abortion-related complications. About 18.5% respondents sought treatment from drug-sellers and 12.3% did not seek any treatment (**data not shown**).

#### Experiences related to current pregnancy

Of 66 pregnant women, the majority confirmed pregnancy via pregnancy strip (51.5%), urine test and skilled provider checkup (10% and 6% respectively). About one-third of the respondents were found from each trimester period of pregnancy, for example, first trimester (0–3 month) (36.4%), 2^nd^ trimester (34.8%) and 3^rd^ trimester (28.8%) respectively. About three-fourth (75.8%) of the respondents planned to continue the pregnancy while about 12% planned to terminate the pregnancy. Of 50 FSWs having a plan to continue the pregnancy, 26% did not visit a healthcare provider. Forty-eight percent of respondents were continuing to practice commercial sex in spite of current pregnancy, with the main reason cited as the regular source of income (Data not shown).

#### Experiences on maternal healthcare

- Antenatal care (ANC) of FSWs who had childbirth: A total of 61 women were found who had childbirth in past one year. About one-quarter of them completed four or more antenatal care visits (ANC), while 8.2% did not have any ANC visits. All but one respondent reported complications during pregnancy; severe weakness (60.7%), excessive bleeding (41.0%), blurry vision (26.2%), and headache (24.6%) were frequently reported **(Table 4)**.

**Table 4:** Antenatal care characteristics of FSWs with a birth in last year.

Characteristics	%
**No of visit to a health provider during pregnancy (n = 61)**	
0	8.2
1	9.8
2	29.5
3	24.6
≧4	27.7
**Type of healthcare providers who were visited (n = 56)**	
Qualified doctors	55.4
Nurse	32.1
Paramedic	44.6
DIC healthcare providers	7.1
Other community healthcare providers (NGO)	10.7
Other (SACMO/HA/FWA)	7.1
**Suffered from any pregnancy complications (n = 61)**	
Severe weakness	60.7
Excessive bleeding	41.0
Blurry vision	26.2
Oedema	24.6
Headache	24.6
Severe anaemia	16.4
Lower abdominal plain	14.8
STI	13.1
Fever	4.9
High blood pressure	6.6
UTI	4.9
Other (eg., Convulsion/breathing problems/uterus prolapsed/diabetes)	9.8
No complication	1.6

- Delivery care of FSWs: Of 61 FSWs having a delivery in last one year, 80.3% delivered within their locality in Dhaka city while 11.5% went to their village or outside Dhaka. Delivery took place at different places such as home/place of living (39.3%), not-for-profit private health facilities (NGO) (27.9%), public health facilities (16.4%) and for-profit private health facilities (16.4%). Three-fourth of the deliveries were normal vaginal delivery and 7 cases (11.5%) underwent C-section. All respondents reported a live birth, with 83.6% of respondents reporting a healthy newborn and 16.4% (10 cases) reported a sick baby. Of the 10 sick babies, five cases (50%) were born with low birth weight, 3 cases were lethargic or unable to feed and 3 had birth defect or birth injury **(Table 5)**.

**Table 5:** Delivery care of FSWs who had a childbirth in last one year (n = 61).

Characteristics	%
**Areas of childbirth**	
Within their locality (In Dhaka city)	80.3
Outside of their locality (In Dhaka city)	8.2
In native village/outside Dhaka city	11.5
**Places of trial for childbirth**	
Home	65.6
Not-for-profit private health facilities (NGO)	32.8
Public health facilities	16.4
For profit private health facilities	16.4
**Places of childbirth**	
Home/ Place of residence	39.3
Not-for-profit private health facilities (NGO)	27.9
Public health facilities	16.4
For profit private health facilities	16.4
**Type of birth attendants**	
Skilled	54.1
Trained traditional birth attendants	11.5
Unskilled	34.4
**Type of delivery**	
Normal vaginal delivery without Episiotomy	75.4
Normal vaginal delivery with Episiotomy	11.5
C-section	11.5
Assisted vaginal delivery (Forcep)	1.6
**Outcome of delivery**	
Live birth	100.0
Stillbirth	0
**Physical condition of the newly born baby**	
Healthy baby	83.6
Sick baby	16.4
**Type of health problems of the newly born baby (n = 10)**	
Low birth weight (<2.5 kg wt)	50.0 (5 cases)
Lethargic/unable to breast-fed	30.0 (3)
Birth defect/birth injury	30.0 (3)
**Type of health providers who provided treatment to the babies**	
Qualified doctors	60.0
Paramedic	30.0
Traditional birth attendant	10.0
**Sex of the babies (n = 61)**	
Boy	50.8
Girl	49.2

- Postnatal care (PNC): The FSWs were asked whether they visited any medical persons for checkup within 42 days after their last delivery. More than a half reported not having a postnatal visit after childbirth. Only about one-quarter of FSWs (23%) reported two or more PNC visits. The main providers of PNC included qualified doctors (56.7%), nurse (33.3%) and paramedics (26.7%) (Data not shown).

#### Reported sexually transmitted infections (STIs) symptoms

About 41.6% (304 cases) of 731 FSWs reported at least one STI symptom in the last one year. Among them, 73.7% and 45.7% reported having vaginal discharge and ulcer in genital area respectively. A small proportion (10.5%) mentioned vaginal itching. Data revealed that 206 of 304 cases (97.4%) sought treatment for STI symptoms. Sixty percent of respondents received treatment from a DIC while 14% visited other NGOs, 11.1% visited public facilities, and 16.9% sought treatment from a drug shop **(Data not shown)**.

### Phase II: Findings from workshop

During the workshop, participants noted the DIC program is supported by international donors, namely the Global Fund, and is not integrated into routine health services. The DIC was established to assist in preventing HIV in target populations (eg., FSWs, transgender), since these populations continue to be excluded from the public health system. The DIC model has been successful at contributing to the prevention of STIs/AIDS in these populations; however, the Global Fund is reducing financial support due to economic growth in Bangladesh over the last several years. One participant cited as-

*“Actually, HIV is spreading through particular lifestyles like unproductive sex by the sex workers, injecting drug users. However, still now our healthcare system is not ready for delivering SRH-related services for FSWs; it’s not a problem only for Bangladesh but for other countries. DIC is the most successful model aimed at STI treatment and HIV prevention. ………. Due to low prevalence in HIV and onwards to middle income country, fund allocation for DIC has been reduced in Bangladesh. Donors are expecting quality services from within the present allocation; however, it’s quite impossible. Still we are working in DIC model but into a modified way. We have reduced the human resource and the coverage households as well”*.

It is expected that the Government of Bangladesh will allocate fund for moving forward the DIC model to prevent STIs/AIDS among this population. However, it will be difficult for the Government to maintain this program, given their lack of involvement to date.

It is feasible to integrate SRH services within existing DIC to ensure access for FSWs; however, this integration would require expansion of existing infrastructure and human resources. One participant noted that this integration was feasible, but would have budget implications. In addition, efforts to ensure quality services would need to be implemented. The participants noted that reorganization of existing DIC services is not currently feasible, however it may be possible to provide referral services for DIC clients in need of SRH services, including information where services are available. In addition, it may be possible for DICs to organize outreach and media campaigns every 3 months to share information. The participant depicted as “*within our existing setup we should provide valid referral link to the patients*. *We should just inform where the particular services are available*. *As well as*, *may arrange campaign in every 3 months*”. In parallel, participants discussed the possibility of an assessment on feasibility of incorporating SRH interventions in existing DICs on staff workload. One participant mentioned potentiality of incorporating SRH services in an ongoing NGO Dhaka-based health service delivery project (NHSDP) such as contraceptive and maternal health services.

The participants also discussed additional barriers to quality implementation of SRH services. For example, one participant noted the Government and other healthcare providers are not well trained to provide services to FSW. She stated:

*“Service providers of DIC are FSW friendly. They are well trained in accommodating a friendly environment whereas; the Government service providers are not well trained to provide services to FSWs. Therefore, Government service providers need an orientation. She asked, why don’t FSWs feel free, though services and medicines are available? Why should we confirm all the services from DIC? Why don’t other facilities? We should answer these questions to solve the problems. Normally, FSWs are seeking treatment as human, not using her identity as sex workers but when their identity is exposed, treatment changed. They are neglected and stigmatized*.*”*

Discussants added that gender equity is included in the Government 5-year operational plan which should extend to FSWs to ensure social inclusion, “*Govt*. *is taking initiatives to ensure gender equity into the 5 year operational plan*. *To ensure gender equity*, *social inclusion of FSWs is needed*, *otherwise the problem will sustain*”. However, promotion of gender equity for FSWs within the formal healthcare system will take time, with advocacy to Government and policy level stakeholders. Sustainable financing of DIC and other FSW SRH programs is another issue and needs to be addressed. One participant expressed this,

*“In the Bangladeshi context it’s quite impossible to expect, govt. will create ideal situation for FSW within formal healthcare system. So, we may fight for such an environment with the govt. and policy level stakeholders. Moreover, the total funding for DIC coming from abroad but funding are squeezing may be vanished in near future. So, we should make them sustainable”*.

The participants validated the findings from Phase 1, and recommended that priority recommendations for policy makers include the importance of supporting sustainable DIC services for FSWs. The final recommendations are summarized in **Table 6**.

**Table 6:** Recommendations identified in workshop.

No	Recommendations
1	Government should take responsibility to provide SRH services for FSWs available, affordable and accountable
2	Conduct orientation meeting and workshop with Government key personnel including national policy makers for acceptability and readiness to support DIC services
3	Test the integration of contraceptive and maternal health services with DIC services within the existing NGO health service delivery project (NHSDP)
4	Implementation of SRH services within DIC will require additional funds, human resources, and infrastructure.
5	Under the auspices of the Government 5-year operational plan for health services, gender equity should be promoted for FSWs within the formal healthcare system
6	International Donors should continue to fund DICs until the Government is prepared to continue DIC services

## Discussion

This study demonstrated higher menstrual regulation, abortion and birth rate among FSWs compared to non-FSWs of reproductive age. The national MR and abortion rate annually was 18 per 1000 women [17] and childbirth status was 20 live births/thousands women [18]. Our findings related to abortion is similar to another recent study by Katz et al where abortion rate among FSWs in Dhaka was 13% to 16% [19]. In addition, there are other studies outside of Bangladesh which have documented life time abortion rates of more than 50% among FSWs, which further corroborate our findings [20–24]. Katz et al demonstrated that 32% to 53% of FSWs sought maternal and child care services; however, they did not note the childbirth rate.

MR by MVA has been implemented in Bangladesh apart of the national family planning program since 1979 for regulation of pregnancies within 8 to 10 weeks after a woman’s missed period. An induced abortion can also be offered to save the life of the woman [25].Tablets (eg., Oxytocin or misoprostol) for early pregnancies (>6 weeks) and D & C for advanced pregnancies (7 to 10 weeks) are standard methods for induced abortion in Bangladesh. Studies indicated that women often used introduced foreign objects into the uterus, abortifacient tablets from drug shops or sought care from unqualified allopathic practitioners for abortion[26]. Another study documented unofficial abortion practices for women who were beyond 10 weeks of pregnancy [27]. In our study, the management of abortion among FSWs reflected the use of these practices. Complications of abortion were reported among more than 50% FSWs indicating potentially inducing abortion after the recommended duration and/or lack of accessible post-abortion care. A study conducted in Uzbekistan reported more than a quarter of FSWs had an abortion during the second trimester (26.8%) [20].Our study further noticed that 35% of respondents reported going to private-for-profit clinics which are costly. Unpublished qualitative data documented that FSWs were charged higher rates for abortion services and healthcare providers treated them differently than non-FSW clients.

In Bangladesh, pregnant women are encouraged to attend at least 4 ANC visits at16 weeks, 24–28 weeks, 32 weeks and 36 weeks of pregnancy[28,29]. Our study findings indicated that 27.7% of FSWs had four or more ANC visits compared to 45.5% of pregnant women in urban areas [8]. Although there have been a few studies [30–32]on FSW’s ANC practices, there is a lack of information on the number of ANC visits or the type of care. In one study in India, FSWs reported HIV testing during pregnancy; other studies by Beckham et al and Willis et al described barriers to ANC services mainly due to discrimination or unfriendly behavior of healthcare providers [30–32]. Our study further added that almost all FSWs reported at least one pregnancy complication.

There was a large variation on proportion of FSWs with a child born as a result of sex work, ranging from 51% in Madagascar, 33.2% in Ethiopia and 8.3% in our study [33]. Geographical variation could explain the different rates among these studies. The proportion of FSWs with home birth (39.3%) was comparable to national surveys where 42.3% of urban residents had a home delivery [8]. In one study in India, 44.5% of FSWs reported a home birth, which is similar to our study [34]. There is scarcity of research articles found which describe childbirth experiences of FSWs in details, therefore, this paper could be useful for the safe motherhood program to understand the situation and consider initiating specific maternal health programs for FSWs. Although there was 100% live birth in our study, several other studies indicated stillbirth (1% to 14%) [34–36]. Although there were no maternal deaths in our study, Willis et al reported in their multi-regional study that 20% of pregnant FSWs died during pregnancy or delivery in last three years [31]. Only other study by Willis et al demonstrated that FSWs often re-started sex work within a few days or weeks after delivery of their child. Suicides and postnatal depression were also reported in this study [31]. To better understand these conditions among FSWs, it is important to include SRH indicators within a sub-sample of FSWs in national surveys.

Unhealthy practices around management of STIs is common in South Asia, such as using traditional healers, herbalists, injection doctors, drug sellers or pharmacist. [37–39]. However, our study demonstrates improved care seeking practices for STI management. The majority of FSWs in our study sought care from DIC, which is similar to a study in Laos where 53% of FSWs reported receiving care from DIC [40]. While care seeking for STIs by male partners of FSWs in Bangladesh was dominated by pharmacies[41], only 16% of FSWs reported seeking care from pharmacies. DICs appear to be successful in providing STI services to FSWs and should be considered as a important program to improve SRH among this population.

The effective contribution of DICs to provide SRH services to vulnerable populations such as FSWs has been noted in other studies in Laos and Canada [40,42]. Another study in Mysore, India documented an increase in care seeking from DICs from 56% at baseline to 89.6% at follow up (more than two years after baseline) and was significantly associated with improvements in condom use and reducing STIs [43]. However, it has been difficult to sustain funding for DIC programs, including problems with renting cost of office space, retaining staff and funding service delivery, medicine, logistic and equipment [44]. Several Indian studies pointed out limited current or future funding opportunities in the area of HIV/AIDS prevention to support DIC [45,46]. The similar issue was raised during discussion in the Workshop of our study. As DICs are the base of health services targeting FSWs with the aim of integrated SRH services, it is imperative to sustain DIC programs, which was prioritized by workshop participants.

The Workshop participants strongly recommended working with Government and other key national stakeholders to provide SRH services for FSWs, operationalizing the Government’s 5-year operational plan for gender equity and social inclusion of all groups. Advocacy and training of health personnel are priority interventions. The Government has already initiated several supporting initiatives such as women friendly hospitals, increasing coverage of HIV testing and services, providing affordable services, introducing law and policies to increase knowledge and tolerance of health workers, eliminating discriminatory provisions in all laws and policies among others[47]. These actions provide a basis for promoting SRH services for FSWs.

The successful integration of family planning/SRH and HIV services are seen in Kenya, Zambia and other African countries [29,46,48]. In a recent study in 2012, Petruney et al addressed the need of developing, implementing and evaluating integration of HIV prevention and family planning services in Asia [49]. Throughout the Workshop, the need for integration of SRH services with existing DIC services was discussed. Other practical suggestions were to collaborate with exiting Government-funded projects (eg., Urban Primary Healthcare Project or UPHCP) or Donor funded projects (eg., NGO Health Service Delivery project or NHSDP) as they are responsible for providing maternal, neonatal, child health-family planning (MNCH-FP) services in urban areas.

This study had some limitations. First, recall or response bias may influence survey responses. The timing of ANC visits was not recorded; therefore, these findings cannot confirm whether these visits were done within the recommended timeframe, although this was not a specific research question. Measuring the psychological state of study women was also not an objective; therefore, it was not covered by this study. Similarly, this study did not record or collect data on the number of children who were given up for adoption; this is why, we cannot assess any relationship between adoption rate and abortion. The sample of FSWs who reported abortion or maternal healthcare experiences was relatively small, and it is difficult to draw concrete conclusions from this sample.

Based on this study, there are several recommendations. First, the high prevalence of abortion among FSWs suggests the need for improving availability of contraceptives to prevent undesired pregnancies. Second, comprehensive information on SRH services for FSWs should be developed and disseminated via static and outreach services. Third, it is important to improve post abortion care information and services to reduce prevalence of post abortion complications. Fourth, as many FSWs continue pregnancy, developing and targeting safe motherhood interventions is essential. Free testing of HIV service provision during antenatal care at health facilities should also be considered. Finally, DICs are successfully providing STIs treatment to FSWs, and this program should be continued, and the possibility of integrated SRH services explored.

## Conclusion

This descriptive exploratory study generates firm recommendations around SRH services for FSWs. Given the high prevalence of reported post abortion complications and low rates of ANC/PNC and delivery care by skilled providers, there is a need for comprehensive SRH intervention and services. Ideally, SRH services should be easily accessible to FSWs through integration with existing DIC. The study also urges policy makers and program implementers to consider FSWs health needs during pregnancy and childbirth to reduce morbidity and mortality.

## Supporting information

S1 Table Survey questionnaire. (DOCX)
