# Supplementary material for: Sexual and reproductive health behaviors of female sex workers in Dhaka, Bangladesh
Source: PLoS One. 2017 Apr 3;12(4):e0174540. doi: 10.1371/journal.pone.0174540 (PMC5378344; doi:10.1371/journal.pone.0174540)
Supplement: S1 Table — (DOCX) [file pone.0174540.s001.docx]

**Respondent ID**

A mixed method study on barriers in reproductive health service delivery for and service utilization by the female sex workers of Bangladesh:Translating findings to policy brief

**Survey Questionnaire**

**(English version)**

Tasnuva Wahed

Student ID: 57791587 53

A data collection tool to use for thesis study for the Degree of

Doctor of Philosophy Program (PhD) in Public Health

Academic Year: 2014

College of Public Health Sciences, Chulalongkorn University, Thailand

**Section-1A: Demography & General Information**

| SI No | Characteristics | Response | Code | Instruction |
| --- | --- | --- | --- | --- |
| 101 | Location of interview | Nakhal para...... ........ ........ ........ ........ ........ ........ 1  Jatrabari........ ........ ........ ........ ........ ........ ........... 2  Chandkherpool ........ ........ ........ ........ ........ ...........**3** |  |  |
| 102 | What is your current age? | __________(completed years) |  |  |
| 103 | What is your occupation? | Sex work 1  Other 2 |  | If code≠1, skip to End |
| 104 | Type of sex worker  (Multiple answers acceptable) | Street-based 1  Hotel based 2  Residence-based 3  other 8 |  |  |
| 105 | Howlong have you been involved with sex works? | …………… years (completed years) |  | Write ‘00’ if <1 year |
| 106 | How many clients do you entertain a day? | _________________(Nos) |  |  |
| 107 | How many times do you entertain sex work a day? | _________________(Nos) |  |  |
| 108 | What is your current marital status? | Married 1  Divorced ………………………………………………2  Widowed………………………………………………3  Separated for one year or more………………………………….………………..4  Husband abandoned away for one year or more…………………………………………………...5  Husband has been living abroad for one year or more…………………………………………………..6  Unmarried…………………………………….……7  Other……………………………………………………8 |  |  |
| 109 | How many years of schooling have you completed? | --------------------------- (in completed years) |  | Write ‘00’ for no education |
| 110 | Where is your dwelling place? | Street……………………………………………………1  Slum…………………………………………………….2  General residence……………………………………….3  Other…………………………………………………….4 |  |  |
| 111 | What is your income from sex work (in BDT) | 1. Daily_____________taka, 2. Monthly___________________taka |  |  |

Section-1B: Obstetric history of a mother

| SI No | Characteristics | Response |  |  | Code | Instruction |
| --- | --- | --- | --- | --- | --- | --- |
| 112 | How many times have you been pregnant in your lifetime? (Gravida) | ------------ | (Nos) |  |  | If answer is 0, skip to 201 |
| 113 | How many times have you delivered a live/dead baby in your lifetime? (Para) | __________ (Nos) |  |  |  |  |
| 114 | How many times did you experience abortion? | __________ (Nos) |  |  |  |  |

Section-2: Contraceptive use

| SI No | Characteristics | Response | Code | Instruction |
| --- | --- | --- | --- | --- |
| 201 | What are the contraceptive methods?  (Multiple answers acceptable) | Oral pill 1  Condom 2  IUD 3  Implant 4  Injectables 5  Female sterilization 6  Male sterilization 7  Natural methods 8  Don’t know 77  Others (Specify) 88 |  |  |
| 202 | What are the contraceptive methods available in your area? (Multiple answers acceptable) | Oral pill 1  Condom 2  IUD 3  Implant 4  Injectables 5  Female sterilization 6  Male sterilization 7  Natural methods 8  Don’t know 77  Others (Specify) 88 |  |  |
| 203 | Where are contraceptive services available? (Multiple answers acceptable) | Community health workers 1  Shops 2  Govt. hospitals/health centres 3  Private for profit clinic/NGOs 4  NGOs clinic/hospital 5  DIC 6  Don’t know 7  Others (Specify) 8 |  |  |
| 204 | Are there any barriers to get contraceptive services?  (Multiple answers acceptable) | Costly/lack of money 1  Not available always /Inconveinient time 2  Do not know where to get 3  Far distance 4  The misbehavior of provider 5  Lack of transport 6  Feeling shameful to get this services 7  Suppliers/Sellers/Service providers do not like to sell or give services 8  There is no barriers 9  Don’t know 77  Others (Specify) 88 |  |  |
| 205 | Are you currently pregnant? | Yes 1  No 2  Don’t know 3 |  | If yes/don’t know, skip to 301 |
| 206 | Please, tell us your current status of using contraceptive methods  (Single answers acceptable) | Oral pill 1  Condom 2  IUD 3  Implant 4  Injectables 5  Female sterilization 6  Do not use anything 7  Natural methods 8  Others (Specify) 88 |  | If code=7, Go to 208 |
| 207 | Where do you collect contraceptive methods from? (Multiple answers acceptable) | Community health workers 1  Shops 2  Govt. hospitals/health centres 3  Private for profit clinic/ hospital 4  NGOs clinic/hospital 5  DIC 6  Others (Specify) 8 |  |  |
| 208 | Did you face any barriers to get contraceptive services? | Yes 1  No 2 |  | If no,skip to 210 |
| 209 | If yes, what types of barriers did you face? (Multiple answers acceptable) | Costly/lack of money 1  Not available always /Inconveinient time 2  Do not know where to get 3  Far distance 4  The misbehavior of provider 5  Lack of transport 6  Feeling shameful to get this services 7  Suppliers/Sellers/Service providers do not like to sell or give services 8  Others (Specify) 88 |  |  |
| 210 | Did you face any barriers to use any contraceptives? | Yes 1  No 2 |  | hw` bv nq, 301 G hvb |
| 211 | If yes, what types of barriers did you face? (Multiple answers acceptable) | Clients’ restriction 1  Did not have knowledge on contraceptives 2  Lack of time to take contraceptive services 3  Others (Specify) 8 |  |  |

# Section-3: Abortion care (Mf©cvZ †mev)

| SI No | Characteristics | | | Response | | | Code | | Instruction |
| --- | --- | --- | --- | --- | --- | --- | --- | --- | --- |
| 301 | Where are the abortion services available in your area? (Multiple answers acceptable) | | a. What are the areas? | | | b. What are the sources?  (*Please, use the code below) | |  |  |
|  |  |  | a1. In this area (in Dhaka)…......................1 | | |  | |  |  |
|  |  |  | a2. Other area (in Dhaka)….......................2 | | |  | |  |  |
|  |  |  | a3.own village/native village/out of Dhaka city ...... ...... ............3 | | |  | |  |  |
|  | *Codes: By skilled providers at own home=1, at skilled providers home =2, specialized hospital=3, Govt Medical College Hospital=4, District hospital=5, Maternal & Child welfareS centre(MCWC)=6, Upazilla Health Complex=7,Union health and family wealth fare centre=8, Union sub-centre=9, Private Medical College Hospital =10, DIC=11, Not-for-profit NGO=12, Private for-profit clinic/hospital=13, Pharmacies=14, Doctor’s chambers=15, at own home by self/family members/neighbor/relatives=16, at own home by unskilled providers=17, at unskilled providers’ home=18, Others (Specify)=19, Don’t know=77 | | | | | | | | |
| 302 | Are there any barriers to get formal health services for MR/Abortion? | | Yes 1  No 2  Don’t know 7 | | | |  | | Skip to 304 |
| 303 | If yes, what types of barriers are there? (Multiple answers acceptable) | | Costly/lack of money 1  Not available always /Inconveinient time 2  Do not know where to get 3  Far distance 4  The misbehavior of provider 5  Lack of transport 6  Feeling shameful to get this services 7  Service providers do not like to sell or give services 8  Others (Specify) 88 | | | |  | |  |
| 304 | Did you experience any abortion within last one year? | | Yes………………………………………………….1  No……………………………………………….…..2 | | | |  | | If no go to next section |
| 305 | Where did you seek care for termination of pregnancy during last time? (Multiple answers acceptable) | | a. What are the areas? | | | b. What are the sources?  (*Please, use the code below) |  | |  |
|  |  |  | a1. In this area (in Dhaka)…......................1 | | |  |  |  |  |
|  |  |  | a2. Other area (in Dhaka)….......................2 | | |  |  |  |  |
|  |  |  | a3.own village/native village/out of Dhaka city ...... ...... ............3 | | |  |  |  |  |
|  | *Codes: By skilled providers at own home=1, at skilled providers home =2, specialized hospital=3, Govt Medical College Hospital=4, District hospital=5, Maternal & Child welfareS centre(MCWC)=6, Upazilla Health Complex=7,Union health and family wealth fare centre=8, Union sub-centre=9, Private Medical College Hospital =10, DIC=11, Not-for-profit NGO=12, Private for-profit clinic/hospital=13, Pharmacies=14, Doctor’s chambers=15, at own home by self/family members/neighbor/relatives=16, at own home by unskilled providers=17, at unskilled providers’ home=18, Others (Specify)=19, Don’t know=77 | | | | | | | | |
| 306 | a. What type of medicine/treatment did you receive for abortion? (Multiple answer possible) | | | | b Who provides this treatment? Anyone else? (Multiple answers acceptable) * use code from below | |  | |  |
| a1. Herbs | | 1= Yes 2=No | | |  | |  | |  |
| a2. Spiritual (*jharfuk/ Pani pora*) | | 1= Yes 2=No | | |  | |  | |  |
| a3. Surgery/operation | | 1= Yes 2=No | | |  | |  | |  |
| a4. Homeopath | | 1= Yes 2=No | | |  | |  | |  |
| a5. Oral allopathic medicine | | 1= Yes 2=No | | |  | |  | |  |
| a6. Saline | | 1= Yes 2=No | | |  | |  | |  |
| a7. Injection | | 1= Yes 2=No | | |  | |  | |  |
| a8. Other (specify)--------------------- | | 1= Yes 2=No | | |  | |  | |  |
| a9. Don’t know | | 1= Yes 2=No | | |  | |  | |  |
| *Codes: Qualified doctor=01 Nurse/midwife =02 FWV=03 MA/SACMO=04 HA=05 FWA=06 /paramedic =07 TTBA=08 TBA=09 DIC’s health providers=10 Village doctor /PC=11 Pharmacist=12 Kobiraj=13 Spiritual healer=14 Homeo doctor=15 own=16, neighbor=17, Other (specify)=88____________________________, Don’t. know=77 | | | | | | | | | |
| 307 | If you take oral allopathic medicine, what were the types of medicine? | | | ÑÑÑÑÑÑÑÑÑÑÑÑÑÑÑÑÑÑÑÑÑÑÑÑÑÑÑ  Don’t know =77  Not applicable =99 | | |  | |  |
| 308 | Did you face any health complications after abortion due to abortion? | | | Yes 1  No 2 | | |  | | Go to 311 |
| 309 | What types of health complications did you face?  (Multiple answer possible) | | | Swollen hands, foot, face 1 | | |  | |  |
|  |  |  |  | Headache 2 | | |  | |  |
|  |  |  |  | fit 3 | | |  | |  |
|  |  |  |  | Excessive bleeding 4 | | |  | |  |
|  |  |  |  | High fever 5 | | |  | |  |
|  |  |  |  | High blood pressure 6 | | |  | |  |
|  |  |  |  | Severe abdominal pain 7 | | |  | |  |
|  |  |  |  | Severe anemia 8 | | |  | |  |
|  |  |  |  | Convulsion 9 | | |  | |  |
|  |  |  |  | Severe weakness 10 | | |  | |  |
|  |  |  |  | blurry of vision 11 | | |  | |  |
|  |  |  |  | Urinari Tract Infection (UTI) 12 | | |  | |  |
|  |  |  |  | STIs 13 | | |  | |  |
|  |  |  |  | Bad smell discharge 14 | | |  | |  |
|  |  |  |  | Breathing problems 15 | | |  | |  |
|  |  |  |  | Uterus prolaps/problems 16 | | |  | |  |
|  |  |  |  | Incomplete abortion 17 | | |  | |  |
|  |  |  |  | Others (Specify) 88 | | |  | |  |
| 310 | Whom did you see for treatment? | | | Qualified doctor 1 | | |  | |  |
|  |  |  |  | Nurse/midwife 2 | | |  | |  |
|  |  |  |  | Family welfare visitors (FWV) 3 | | |  | |  |
|  |  |  |  | MA/SACMO 4 | | |  | |  |
|  |  |  |  | Health Assistant (HA) 5 | | |  | |  |
|  |  |  |  | Family welfare assistant (FWA) 6 | | |  | |  |
|  |  |  |  | paramedic 7 | | |  | |  |
|  |  |  |  | DIC’s workers 8 | | |  | |  |
|  |  |  |  | Other Community health workers 9 | | |  | |  |
|  |  |  |  | Trained birth attendants (TTBA) 10 | | |  | |  |
|  |  |  |  | Traditional birth attendants (TBA) 11 | | |  | |  |
|  |  |  |  | BRAC’s UBA 12 | | |  | |  |
|  |  |  |  | Hospital’s aya 13 | | |  | |  |
|  |  |  |  | Pharmacist 14 | | |  | |  |
|  |  |  |  | Kobiraj 15 | | |  | |  |
|  |  |  |  | Spiritual healers 16 | | |  | |  |
|  |  |  |  | Homeo doctor 17 | | |  | |  |
|  |  |  |  | Relatives 18 | | |  | |  |
|  |  |  |  | Neighbour 19 | | |  | |  |
|  |  |  |  | Brokers 20 | | |  | |  |
|  |  |  |  | Others (Specify) 88 | | |  | |  |
|  |  |  |  | No one 21 | | |  | |  |
| 311 | Please, show your health card if you have any? (abortion related)  (Single response) | | | Have and showed card…………………………….…1  Have not card…………………………………….……...2  Have card but cannot show at the time of interview………………………………….………………..3  Other…………………………………………………………4 | | |  | |  |
| 312 | Did you face any barriers to get formal health services? | | | Yes………………………………………….………………...1  No…………………………………………………….….…...2 | | |  | | If no, skip to 401 |
| 313 | If yes, what types of barriers did you face? (Multiple answers acceptable) | | | Costly/lack of money 1  Not available always /Inconveinient time 2  Do not know where to get 3  Far distance 4  The misbehavior of provider 5  Lack of transport 6  Feeling shameful to get this services 7  Service providers do not like to sell or give services 8  Others (Specify) 88 | | |  | |  |

**Section-4: Care seeking of women who is currently pregnant**

Not applicable=99

****If respondent is not currently pregnant, please, circle the code ’99**

**in next box and’skip to 501**

| SI No | Characteristics | Response | | | Code | Instruction |
| --- | --- | --- | --- | --- | --- | --- |
| 401 | How did you confirm your pregnancy?  (Multiple answers acceptable) | Urine test………………….…………………………….….1  Strip …………………………………….………………….…2  Self-perceptions/perceptions of family members/TBAs……………………………………………3  From skilled health providers...................... 4  Others (Specify) ………………………………….8 | | |  |  |
| 402 | Please, show your health card if you have any? (pregnancy related) | Have and showed card……………………………….1  Have not card…………………………….……………...2  Have card but cannot show at the time of interview……………………………….……………….....3  Other………………………………………………….……..8 | | |  |  |
| 403 | What is your current stage of pregnancy | 1^st^ trimester (<3months pregnancy) ….……..….1  2^nd^ trimester (3-6 months pregnancy…………...2  3^rd^ trimester (>6months pregnancy)……………..3 | | |  |  |
| 404 | What is your plan with current pregnancy? | No plan 1  Continue pregnancy up to childbirth 2  Termination of pregnancy 3 | | |  | If code=1,2, skip to 406 |
| 405 | Where will you seek care for termination of pregnancy? (Multiple answers acceptable) | a. What are the areas? | b. What are the sources?  (*Please, use the code below) | |  | Skip to next section after getting anser of q405 |
|  |  | a1. In this area (in Dhaka)….....................1 |  | |  |  |
|  |  | a2. Other area (in Dhaka)….....................2 |  | |  |  |
|  |  | a3.own village/native village/out of Dhaka city ...... ...... ............3 |  | |  |  |
|  | *Codes: By skilled providers at own home=1, at skilled providers home =2, specialized hospital=3, Govt Medical College Hospital=4, District hospital=5, Maternal & Child welfareS centre(MCWC)=6, Upazilla Health Complex=7,Union health and family wealth fare centre=8, Union sub-centre=9, Private Medical College Hospital =10, DIC=11, Not-for-profit NGO=12, Private for-profit clinic/hospital=13, Pharmacies=14, Doctor’s chambers=15, at own home by self/family members/neighbor/relatives=16, at own home by unskilled providers=17, at unskilled providers’ home=18, Others (Specify)=19, Don’t know=77 | | | | | |
| 406 | Did you visit any health providers after being pregnant? | Yes………………………………………………………………1  No……………………………………………………………….2 | | |  | If no, skip to 410 |
| 407 | How many times did you receive medical checkup after being pregnant? | a. . As a part of regular check up  ____________( Nos) | | b. For illness  ________( Nos) |  |  |
| 408 | Whom did you see? Anyone else?  (Multiple answers acceptable) | Qualified doctor 1 | | |  |  |
|  |  | Nurse/midwife 2 | | |  |  |
|  |  | Family welfare visitors (FWV) 3 | | |  |  |
|  |  | MA/SACMO 4 | | |  |  |
|  |  | Health Assistant (HA) 5 | | |  |  |
|  |  | Family welfare assistant (FWA) 6 | | |  |  |
|  |  | paramedic 7 | | |  |  |
|  |  | DIC’s outreach workers 8 | | |  |  |
|  |  | Other Community health workers 9 | | |  |  |
|  |  | Trained/untrained birth attendants (TTBA) 10 | | |  |  |
|  |  | Others (Specify) 88 | | |  |  |
| 409 | Where did you receive the medical checkup from?  (Multiple answers acceptable) | a. What are the areas? | b. What are the sources?  (*Please, use the code below) | |  |  |
|  |  | a1. In this area (in Dhaka)….....................1 |  | |  |  |
|  |  | a2. Other area (in Dhaka)….....................2 |  | |  |  |
|  |  | a3.own village/native village/out of Dhaka city ...... ...... ............3 |  | |  |  |
|  | *Codes: By skilled providers at own home=1, at skilled providers home =2, specialized hospital=3, Govt Medical College Hospital=4, District hospital=5, Maternal & Child welfareS centre(MCWC)=6, Upazilla Health Complex=7,Union health and family wealth fare centre=8, Union sub-centre=9, Private Medical College Hospital =10, DIC=11, Not-for-profit NGO=12, Private for-profit clinic/hospital=13, Pharmacies=14, Doctor’s chambers=15, at own home by self/family members/neighbor/relatives=16, at own home by unskilled providers=17, at unskilled providers’ home=18, Others (Specify)=19, Don’t know=77 | | | | | |
| 410 | During your current pregnancy, did you suffer from any of the following problems?  (Multiple answers acceptable) | Swollen hands, foot, face 1 | | |  |  |
|  |  | Headache 2 | | |  |  |
|  |  | Convulsion /fit 3 | | |  |  |
|  |  | Per vaginal watering before 37 weeks of pregnancy 4 | | |  |  |
|  |  | Per vaginal bleeding (with or without abdominal pain) 5 | | |  |  |
|  |  | Fever for >/= 3 days 6 | | |  |  |
|  |  | High blood pressure 7 | | |  |  |
|  |  | Severe vomiting 8 | | |  |  |
|  |  | Severe abdominal pain 9 | | |  |  |
|  |  | Severe anemia 10 | | |  |  |
|  |  | Jaundice 11 | | |  |  |
|  |  | Inadequate weight gain after first 3 months 12 | | |  |  |
|  |  | Severe weakness 13 | | |  |  |
|  |  | blurry of vision 14 | | |  |  |
|  |  | Urinari Tract Infection (UTI) 15 | | |  |  |
|  |  | STIs 16 | | |  |  |
|  |  | No complications 17 | | |  |  |
|  |  | Others (Specify) 88 | | |  |  |
| 411 | Where is you plan to deliver the child? (Single answer acceptable) | Own house 1  Provider’s house 2  Specialized hospital 3  Medical College Hospital (Public) 4  District hospital 5  MCWC 6  Upazilla Health Complex (UHC) 7  Union Health & Family Welfare Center (UH& FWC)ª 8  Union Sub-Center (USC/RD)ª 9  Medical College Hospital (Private) 10  DIC 11  Not-for-profit (NGO) 12  For-profit (Private)clinic/ hospital 13  No plan 14  Others (Specify) 88 | | |  |  |
| 412 | Did you face any barriers to get formal health services? | Yes…………………………………………………..……...1  No……………………………………………….…………..2 | | |  | If no, skip to 414 |
| 413 | If yes, what types of barriers did you face? | Costly/lack of money 1  Not available always /Inconveinient time 2  Do not know where to get 3  Far distance 4  The misbehavior of provider 5  Lack of transport 6  Feeling shameful to get this services 7  Service providers do not like to sell or give services 8  Others (Specify) 88 | | |  |  |
| 414 | Inspite of having knowledge about your current pregnancy, are you still continuing your sex work? | Yes………………………………………………………………1  No……………………………………………………………….2 | | |  | If no, skip to 501 |
| 415 | If yes, what are the reasons behind it? (Multiple answers acceptable) | Willingly/as a regular income source..............1  Forced by clients..............................................2  Others (Specify) ................................................8 | | |  |  |

**Section-5A: Maternal healthcare of mother who is not currently pregnant**

Not applicable=99

****If respondent is currently pregnant, please, circle the code ’99**

**in next box and’skip to 505**

| SI No | Characteristics | Response | Code | Instruction |
| --- | --- | --- | --- | --- |
| 501 | What type of maternal healthcare services are available in your area (Multiple answers acceptable) | Basic antenatal care by qualified health providers.................1  Normal delivery care by qualified health providers……….……2  Basic postnatal care by qualified health providers ……..……...3  Emergency obstetric care………………………..…………..…..…..……4  Diagnostic services…………………………………………..………………..5  Services by TBAs…………………………………………..………………..….6  Services by Village doctors/pharmacist………………….…...........7  Services by spiritual healers…………………………..….………...…….8  No services available……………………………………………....…………9  Others (Specify)…………………………………………………….88  Don’t know…………………………………………77 |  |  |
| 502 | Where are the maternal health services available in your area? (Multiple answers acceptable) | Own house by skilled providers………………………….……………..1  Skilled provider’s house…….…………………………..………………….2  Specialized hospital……………………………………….…….……………3  Medical College Hospital (Public)……………………..……….……….4  District hospital…………………………………………….…………………..5  MCWC………………………………………………………….…………..………6  Upazilla Health Complex (UHC)……………………….…………………7  Union Health & Family Welfare Center (UH& FWC)…………………………………………………………………….…………..8  Union Sub-Center (USC/RD)……………………………………..……….9  Medical College Hospital (Private)…………………………………...10  DIC…………………………………………………………………………………..11  Not-for-profit (NGO)………………………………………………………..12  For-profit (Private)…………………………………………………………..13  Own house by own/ familymembers/ neighbor/relatives ..14  Own house by unskilled providers……………………………………15  Unskilled provider’s house……...……………………………………….16  Drug shop/pharmacy………………………………..……………………..17  Others (Specify)……………………………………..88  Don’t know………………………………………………………………………77 |  |  |
| 503 | Did you face any barriers to get formal health services? | Yes………………………………………………………….……...1  No………………………………………………………….….…...2 |  | If no, skip to 505 |
| 504 | If yes, what types of barriers did you face? | Costly/lack of money 1  Not available always /Inconveinient time 2  Do not know where to get 3  Far distance 4  The misbehavior of provider 5  Lack of transport 6  Feeling shameful to get this services 7  Service providers do not like to sell or give services 8  Others (Specify) 88 |  |  |

**Section-5B: Maternal healthcare of mother who had childbirth within last year**

| SI No | Characteristics | Response | | | | Code | | Instruction |
| --- | --- | --- | --- | --- | --- | --- | --- | --- |
| 505 | Did you have a childbirth within last one year | Yes………………………………………………...1  No…………………………………………….…...2 | | | |  | | If no skip to 601 |
| 506 | Did you visit any health providers after being pregnant? | Yes…………………………………………………1  No………………………………………………….2 | | | |  | | If no skip to 510 |
| 507 | How many times did you receive medical checkup after being pregnant? | a. . As a part of regular check up  ______________( Nos) | | b. For illness  ____________( Nos) | |  | |  |
| 508 | Whom did you see? Anyone else?  (Multiple answers acceptable) | Qualified doctor 1 | | | |  | |  |
|  |  | Nurse/midwife 2 | | | |  | |  |
|  |  | Family welfare visitors (FWV) 3 | | | |  | |  |
|  |  | MA/SACMO 4 | | | |  | |  |
|  |  | Health Assistant (HA) 5 | | | |  | |  |
|  |  | Family welfare assistant (FWA) 6 | | | |  | |  |
|  |  | Paramedic 7 | | | |  | |  |
|  |  | DIC’s outreach workers 8 | | | |  | |  |
|  |  | Other Community health workers 9 | | | |  | |  |
|  |  | Trained/untrained birth attendants (TTBA) 10 | | | |  | |  |
|  |  | Others (Specify) 88 | | | |  | |  |
| 509 | Where did you receive the medical checkup from?  (Multiple answers acceptable) | | a. What are the areas? | | b. What are the sources?  (*Please, use the code below) | |  |  |
|  |  |  | a1. In this area (in Dhaka)….....................1 | |  | |  |  |
|  |  |  | a2. Other area (in Dhaka)….....................2 | |  | |  |  |
|  |  |  | a3.own village/native village/out of Dhaka city ...... ...... ............3 | |  | |  |  |
|  | *Codes: By skilled providers at own home=1, at skilled providers home =2, specialized hospital=3, Govt Medical College Hospital=4, District hospital=5, Maternal & Child welfareS centre(MCWC)=6, Upazilla Health Complex=7,Union health and family wealth fare centre=8, Union sub-centre=9, Private Medical College Hospital =10, DIC=11, Not-for-profit NGO=12, Private for-profit clinic/hospital=13, Pharmacies=14, Doctor’s chambers=15, at own home by self/family members/neighbor/relatives=16, at own home by unskilled providers=17, at unskilled providers’ home=18, Others (Specify)=19, Don’t know=77 | | | | | | | |
| 510 | During your last pregnancy, did you suffer from any of the following problems?  (Multiple answers acceptable) | Swollen hands, foot, face 1 | | | |  | |  |
|  |  | Headache 2 | | | |  | |  |
|  |  | Convulsion /fit 3 | | | |  | |  |
|  |  | Per vaginal watering before 37 weeks of pregnancy 4 | | | |  | |  |
|  |  | Per vaginal bleeding (with or without abdominal pain) 5 | | | |  | |  |
|  |  | Fever for >/= 3 days 6 | | | |  | |  |
|  |  | High blood pressure 7 | | | |  | |  |
|  |  | Severe vomiting 8 | | | |  | |  |
|  |  | Severe abdominal pain 9 | | | |  | |  |
|  |  | Severe anemia 10 | | | |  | |  |
|  |  | Jaundice 11 | | | |  | |  |
|  |  | Inadequate weight gain after first 3 months 12 | | | |  | |  |
|  |  | Severe weakness 13 | | | |  | |  |
|  |  | Blurry of vision 14 | | | |  | |  |
|  |  | Urinari Tract Infection (UTI) 15 | | | |  | |  |
|  |  | STIs 16 | | | |  | |  |
|  |  | Diabets 17 | | | |  | |  |
|  |  | Breathing problems 18 | | | |  | |  |
|  |  | Uterus prolaps/problems 19 | | | |  | |  |
|  |  | Others (Specify) 88 | | | |  | |  |
|  | **Section-5B2: Childbirth experiences** | | | | |  | |  |
| 511 | On which month of pregnancy did you deliver your child? | __________ ^th^month | | | |  | |  |
| 512 | What was the place of trial for delivery? (Multiple answers acceptable) | Own house …………………………….……………………..1  Skilled provider’s house…….……….………………….2  Specialized hospital………………………….……………3  Medical College Hospital (Public)………….……….4  District hospital……………………………………………..5  MCWC…………………………………………………..………6  Upazilla Health Complex (UHC)………………………7  Union Health & Family Welfare Center (UH& FWC)……………………………………………………………..8  Union Sub-Center (USC/RD)…………………..……….9  Medical College Hospital (Private)………………...10  DIC………………………………………………………………..11  Not-for-profit (NGO)……………………………………..12  For-profit (Private)………………………………………..13  Others (Specify) …………………………………….88 | | | |  | |  |
| 513 | Where did you give birth to (NAME)? (Single answer acceptable) | | a. What are the areas? | | b. What are the sources?(*Please, use the code below) | |  |  |
|  |  |  | a1. In this area (in Dhaka)….....................1 | |  | |  |  |
|  |  |  | a2. Other area (in Dhaka)….....................2 | |  | |  |  |
|  |  |  | a3.own village/native village/out of Dhaka city ...... ...... ............3 | |  | |  |  |
|  | *Codes: By skilled providers at own home=1, at skilled providers home =2, specialized hospital=3, Govt Medical College Hospital=4, District hospital=5, Maternal & Child welfareS centre(MCWC)=6, Upazilla Health Complex=7,Union health and family wealth fare centre=8, Union sub-centre=9, Private Medical College Hospital =10, DIC=11, Not-for-profit NGO=12, Private for-profit clinic/hospital=13, Pharmacies=14, Doctor’s chambers=15, at own home by self/family members/neighbor/relatives=16, at own home by unskilled providers=17, at unskilled providers’ home=18, Others (Specify)=19, Don’t know=77 | | | | | | | |
| 514 | What was the type of delivery? (Single answer acceptable) | Normal delivery (vaginal) with episiotomy……..1  Normal delivery with out episiotomy…………....2  Assisted normal delivery (Forcep)……………….3  Assisted normal delivery (Ventose)……………..4  C/S delivery…………………………………………..……5 | | | | \|  \| \| --- \| | |  |
| 515 | Who assisted during delivery?  (Multiple answers acceptable) | Qualified doctor 1 | | | |  | |  |
|  |  | Nurse/midwife 2 | | | |  | |  |
|  |  | Family welfare visitors (FWV) 3 | | | |  | |  |
|  |  | MA/SACMO 4 | | | |  | |  |
|  |  | Health Assistant (HA) 5 | | | |  | |  |
|  |  | Family welfare assistant (FWA) 6 | | | |  | |  |
|  |  | paramedic 7 | | | |  | |  |
|  |  | DIC’s workers 8 | | | |  | |  |
|  |  | Other Community health workers 9 | | | |  | |  |
|  |  | Trained birth attendants (TTBA) 10 | | | |  | |  |
|  |  | Traditional birth attendants (TBA) 11 | | | |  | |  |
|  |  | BRAC’s UBA 12 | | | |  | |  |
|  |  | Hospital’s aya 13 | | | |  | |  |
|  |  | Pharmacist 14 | | | |  | |  |
|  |  | Kobiraj 15 | | | |  | |  |
|  |  | Spiritual healers 16 | | | |  | |  |
|  |  | Homeo doctor 17 | | | |  | |  |
|  |  | Relatives 18 | | | |  | |  |
|  |  | Neighbour 19 | | | |  | |  |
|  |  | Brokers 20 | | | |  | |  |
|  |  | Others (Specify) 88 | | | |  | |  |
|  |  | No one 21 | | | |  | |  |
| 516 | Who did conduct the delivery/ Who caught the baby actually? | Qualified doctor 1 | | | |  | |  |
|  |  | Nurse/midwife 2 | | | |  | |  |
|  |  | Family welfare visitors (FWV) 3 | | | |  | |  |
|  |  | MA/SACMO 4 | | | |  | |  |
|  |  | Health Assistant (HA) 5 | | | |  | |  |
|  |  | Family welfare assistant (FWA) 6 | | | |  | |  |
|  |  | paramedic 7 | | | |  | |  |
|  |  | DIC’s workers 8 | | | |  | |  |
|  |  | Other Community health workers 9 | | | |  | |  |
|  |  | Trained birth attendants (TTBA) 10 | | | |  | |  |
|  |  | Traditional birth attendants (TBA) 11 | | | |  | |  |
|  |  | BRAC’s UBA 12 | | | |  | |  |
|  |  | Hospital’s aya 13 | | | |  | |  |
|  |  | Pharmacist 14 | | | |  | |  |
|  |  | Kobiraj 15 | | | |  | |  |
|  |  | Spiritual healers 16 | | | |  | |  |
|  |  | Homeo doctor 17 | | | |  | |  |
|  |  | Relatives 18 | | | |  | |  |
|  |  | Neighbour 19 | | | |  | |  |
|  |  | Others (Specify) 88 | | | |  | |  |
|  |  | No one 20 | | | |  | |  |
| 517 | What is the sex of your last delivered child? (in case of twin baby, Multiple answer possible) | Boy 1  Girl 2 | | | |  | |  |
| 518 | What was the outcome of delivery? | Live birth (Single Baby) 1  Live births (Twin Baby) 2  Still birth + Live birth (Twin Baby) 3  Still birth (single baby) 4  Still birth (twin baby) 5 | | | |  | | Go to 522 |
| 519 | What was the physical condition of the newborn? (in case of twin baby, Multiple answer possible) | Healthy baby 1  Sick baby 2  Others (Specify) 3 | | | |  | | Go to 522 |
| 520 | If sick baby, what type of health problems did your baby suffer? (GKvwaK DËi MÖnb †hvM¨) | Birth Asphyxia…………………………………………….…...1Very Low birth weight (</= 1.5 kg)…………………...2Low birth weight (> 1.5 kg- <2.5 kg)………….………3Fever………………………………………………………………..4Unable to feed…………………………………..………………5Less movement of hands and legs/Lethargy………6Convulsions/fits……………………………….………………7Umbilical infection……………………….……………………8Eye infection……………………………………………………..9Skin infection………………………………..………………...10Jaundice……………………………………………….…………11Congenital anomalies ……………………………………...12Birth injury…………………………………………..………....13 Don’t know as I sold/gave the baby to other after birth..........................................................................14  Others (Specify)……………………………… ......................................................88 | | | |  | |  |
| 521 | Who provided treatment for these complications? (Multiple answers acceptable) | Qualified doctor 1 | | | |  | |  |
|  |  | Nurse/midwife 2 | | | |  | |  |
|  |  | Family welfare visitors (FWV) 3 | | | |  | |  |
|  |  | MA/SACMO 4 | | | |  | |  |
|  |  | Health Assistant (HA) 5 | | | |  | |  |
|  |  | Family welfare assistant (FWA) 6 | | | |  | |  |
|  |  | paramedic 7 | | | |  | |  |
|  |  | DIC’s workers 8 | | | |  | |  |
|  |  | Other Community health workers 9 | | | |  | |  |
|  |  | Trained birth attendants (TTBA) 10 | | | |  | |  |
|  |  | Traditional birth attendants (TBA) 11 | | | |  | |  |
|  |  | BRAC’s UBA 12 | | | |  | |  |
|  |  | Hospital’s aya 13 | | | |  | |  |
|  |  | Pharmacist 14 | | | |  | |  |
|  |  | Kobiraj 15 | | | |  | |  |
|  |  | Spiritual healers 16 | | | |  | |  |
|  |  | Homeo doctor 17 | | | |  | |  |
|  |  | Relatives 18 | | | |  | |  |
|  |  | Neighbour 19 | | | |  | |  |
|  |  | Others (Specify) 88 | | | |  | |  |
|  | **Section-5B3: Post natal care** | | | | |  | |  |
| 522 | After (NAME) was born, did any medical persons check on your health? | Yes………………………………………………………………………….1  No………………………………………………………………..………...2 | | | |  | | if no then skip to 601 |
| 523 | How many days or weeks after the delivery did the first check take place? | a. _________Days after delivery  Don’t know=999 | | | |  | | Record ‘00’ days if same day |
| 524 | Who checked on your health at that time?  Anyone else?  (Multiple answer possible) | Qualified doctor 1 | | | |  | |  |
|  |  | Nurse/midwife 2 | | | |  | |  |
|  |  | Family welfare visitors (FWV) 3 | | | |  | |  |
|  |  | MA/SACMO 4 | | | |  | |  |
|  |  | Health Assistant (HA) 5 | | | |  | |  |
|  |  | Family welfare assistant (FWA) 6 | | | |  | |  |
|  |  | Paramedic 7 | | | |  | |  |
|  |  | BRAC’s SK/SS 8 | | | |  | |  |
|  |  | Others (Specify) 88 | | | |  | |  |
| 525 | How many times did you receive medical checkup for your health after your delivery? | __________Nos | | | |  | |  |
| 526 | In the first 6 weeks after birth of (NAME), did you have any of the following problems? | Fever 1 | | | |  | | If answer is Õ15Õ Go to Chapter 6 |
|  |  | Excessive bleeding 2 | | | |  | |  |
|  |  | Foul smelling discharge 3 | | | |  | |  |
|  |  | Severe pain in the lower abdomen 4 | | | |  | |  |
|  |  | Convulsion/fit 5 | | | |  | |  |
|  |  | Wound infection 6 | | | |  | |  |
|  |  | Pain and burning sensation during micturition 7 | | | |  | |  |
|  |  | Severe anemia 8 | | | |  | |  |
|  |  | Respiratory distress 9 | | | |  | |  |
|  |  | Passage of urine or stool per vagina 10 | | | |  | |  |
|  |  | Tender breast and swelling with redness 11 | | | |  | |  |
|  |  | Diarrhoea/ vomiting 12 | | | |  | |  |
|  |  | Pain in stitch / wound 13 | | | |  | |  |
|  |  | Severe weakness 14 | | | |  | |  |
|  |  | Others (Specify) 88 | | | |  | |  |
|  |  | No complications 15 | | | |  | |  |
| 527 | Who provided treatment for these complications?  (Multiple answers acceptable) | Qualified doctor 1 | | | |  | |  |
|  |  | Nurse/midwife 2 | | | |  | |  |
|  |  | Family welfare visitors (FWV) 3 | | | |  | |  |
|  |  | MA/SACMO 4 | | | |  | |  |
|  |  | Health Assistant (HA) 5 | | | |  | |  |
|  |  | Family welfare assistant (FWA) 6 | | | |  | |  |
|  |  | paramedic 7 | | | |  | |  |
|  |  | DIC’s workers 8 | | | |  | |  |
|  |  | Other Community health workers 9 | | | |  | |  |
|  |  | Trained birth attendants (TTBA) 10 | | | |  | |  |
|  |  | Traditional birth attendants (TBA) 11 | | | |  | |  |
|  |  | BRAC’s UBA 12 | | | |  | |  |
|  |  | Hospital’s aya 13 | | | |  | |  |
|  |  | Pharmacist 14 | | | |  | |  |
|  |  | Kobiraj 15 | | | |  | |  |
|  |  | Spiritual healers 16 | | | |  | |  |
|  |  | Homeo doctor 17 | | | |  | |  |
|  |  | Relatives 18 | | | |  | |  |
|  |  | Neighbour 19 | | | |  | |  |
|  |  | Others (Specify) 88 | | | |  | |  |
| 528 | Did you face any barriers to get formal health services? | Yes…………………………………………………...1  No……………………………………………….…...2 | | | |  | | If no skip to 601 |
| 529 | If yes, what types of barriers did you face? | Costly/lack of money 1  Not available always /Inconveinient time 2  Do not know where to get 3  Far distance 4  The misbehavior of provider 5  Lack of transport 6  Feeling shameful to get this services 7  Service providers do not like to sell or give services 8  Others (Specify) 88 | | | |  | |  |

**Section-6: STI/HIV related experiences**

| SI No | Characteristics | | | Response | | | | Code | | Instruction |
| --- | --- | --- | --- | --- | --- | --- | --- | --- | --- | --- |
| 601 | Did you experience any STDs related health problems within last one year? | | | Yes----------------------------------------------1  No-----------------------------------------------2 | | | |  | | If no,skip to 609 |
| 602 | What were those problems? (Multiple answers acceptable) | | | Vaginal discharge………………………….…………1Pelvic inflammatory disease………………….....2Urinary tract infection…………………………….3 Gonorrhoea…………………………………………….4Syphilis………………………………………..…………5Inguinal swelling…………………………………….6 Others (Specify)………………………..8 | | | |  | |  |
| 603 | | Where did you receive the medical checkup from?  (Multiple answers acceptable) | | a. What are the areas? | | | b. What are the sources?  (*Please, use the code below) | |  |  |
|  |  |  |  | a1. In this area (in Dhaka)….....................1 | | |  | |  |  |
|  |  |  |  | a2. Other area (in Dhaka)….....................2 | | |  | |  |  |
|  |  |  |  | a3.own village/native village/out of Dhaka city ...... ...... ............3 | | |  | |  |  |
|  |  | *Codes: By skilled providers at own home=1, at skilled providers home =2, specialized hospital=3, Govt Medical College Hospital=4, District hospital=5, Maternal & Child welfareS centre(MCWC)=6, Upazilla Health Complex=7,Union health and family wealth fare centre=8, Union sub-centre=9, Private Medical College Hospital =10, DIC=11, Not-for-profit NGO=12, Private for-profit clinic/hospital=13, Pharmacies=14, Doctor’s chambers=15, at own home by self/family members/neighbor/relatives=16, at own home by unskilled providers=17, at unskilled providers’ home=18, Others (Specify)=19, Don’t know=77 | | | | | | | | |
| 604 | | 1. What type of medicine/ treatment did you receive for STIs? (Multiple answer possible) | | | | b. Who provides this treatment? Anyone else? (Multiple answers acceptable) * use code from below | |  | |  |
| a1. Herbs | | | 1= Yes 2=No | | |  | |  | |  |
| a2. Spiritual (*jharfuk/ Pani pora*) | | | 1= Yes 2=No | | |  | |  | |  |
| a3. Surgery/operation | | | 1= Yes 2=No | | |  | |  | |  |
| a4. Homeopath | | | 1= Yes 2=No | | |  | |  | |  |
| a5. Oral allopathic medicine | | | 1= Yes 2=No | | |  | |  | |  |
| a6. Saline | | | 1= Yes 2=No | | |  | |  | |  |
| a7. Injection | | | 1= Yes 2=No | | |  | |  | |  |
| a8. Other (specify)--------------- | | | 1= Yes 2=No | | |  | |  | |  |
| a9. Don’t know | | | 1= Yes 2=No | | |  | |  | |  |
| *Codes: Qualified doctor=01 Nurse/midwife =02 FWV=03 MA/SACMO=04 HA=05 FWA=06 /paramedic =07 TTBA=08 TBA=09 DIC’s health providers=10 Village doctor /PC=11 Pharmacist=12 Kobiraj=13 Spiritual healer=14 Homeo doctor=15 own=16, neighbor=17, Other (specify)=88____________________________, Don’t. know=77 | | | | | | | |  | |  |
| 605 | If you take oral allopathic medicine, what were the types of medicine? | | | | ÑÑÑÑÑÑÑÑÑÑÑÑÑÑÑÑÑÑÑÑÑÑÑÑÑÑÑ  Don’t know =77  Not applicable =99 | | |  | |  |
| 606 | Please, show your health card if you have any? (MR/Abortion related)  (Single response) | | | | Have and showed card…………………………….…1  Have not card…………………………….……………...2  Have card but cannot show at the time of interview……………………………….…………………..3  Other……………………………….…………………………8 | | |  | |  |
| 607 | Did you face any barriers to get formal health services? | | | | Yes……………………………………………………….…...1  No…………………………………………………….….…...2 | | |  | | If no,skip to 609 |
| 608 | If yes, what types of barriers did you face? (Multiple answers acceptable) | | | | Costly/lack of money 1  Not available always /Inconveinient time 2  Do not know where to get 3  Far distance 4  The misbehavior of provider 5  Lack of transport 6  Feeling shameful to get this services 7  Service providers do not like to sell or give services 8  Others (Specify) 88 | | |  | |  |
| 609 | Have you ever heard on HIV/AIDS? | | | | Yes…………………….……………………..……………...1  No…………………………………………..……….……....2 | | |  | | If no,skip to 701 |
| 610 | Where are the STI/AIDs services available? | | | | Own house by skilled providers………………………….………..1  Skilled provider’s house…….……….………………….…………….2  Specialized hospital…………………………………….….……………3  Medical College Hospital (Public)…………………….….……….4  District hospital………………………………………….………………..5  MCWC……………………………………………………….………..………6  Upazilla Health Complex (UHC)……………………….……………7  Union Health & Family Welfare Center (UH& FWC) ……..8  Union Sub-Center (USC/RD)………………………………..……….9  Medical College Hospital (Private)……………………………...10  DIC……………………………………………………………………………..11  Not-for-profit (NGO)…………………………………………………..12  For-profit (Private)……………………………………………………..13  Own house by own/familymembers/neighbor/relatives..……………….…14  Own house by unskilled providers………………………………15  Unskilled provider’s house……...………………………………….16  Drug shop/pharmacy………………………………..………………..17  Others (Specify) …………………………………88  Don’t know 77 | | |  | |  |
| 611 | Are there any barriers to get formal health services for HIV/AIDs? | | | | Yes…………………………………………….……………...1  No…………………………………………….………….…...2 | | |  | | If no,skip to 701 |
| 612 | If yes, what types of barriers are there? (Multiple answers acceptable) | | | | Costly/lack of money 1  Not available always /Inconveinient time 2  Do not know where to get 3  Far distance 4  The misbehavior of provider 5  Lack of transport 6  Feeling shameful to get this services 7  Service providers do not like to sell or give services 8  Everyone hate us………………………………...9  Others (Specify) 88  Don’t know 77 | | |  | |  |

**Section 7: Referral care**

| SI No | Characteristics | | Response | | Code | Instruction |
| --- | --- | --- | --- | --- | --- | --- |
| 701 | Were you referred to any health facility for any type of SRH services? | | Yes………………………………………………….. 1  No ……………………………………………………2 | |  | If no,skip to 801 |
| 702 | a. What were the causes of referral from home? (Multiple answer possible) | | b What were the specific causes of referral? Anything else? (open-ended question) | c. Total cost of treatment (in taka) |  |  |
| a1. Contraceptive use-related problem | | 1= Yes 2=No |  |  |  |  |
| a2. Abortion related problems | | 1= Yes 2=No |  |  |  |  |
| a3. Maternal health problem | | 1= Yes 2=No |  |  |  |  |
| a4. STI/HIV related problems | | 1= Yes 2=No |  |  |  |  |
| a5. Other, specify____________ | | 1= Yes 2=No |  |  |  |  |
| 703 | How many referral facilities do you touch for treatment? | | ___________ (Nos) | |  |  |
| 704 | Who made the referral? (Multiple answer possible) | | Qualified doctor 1 | |  |  |
|  |  |  | Nurse/midwife 2 | |  |  |
|  |  |  | Family welfare visitors (FWV) 3 | |  |  |
|  |  |  | MA/SACMO 4 | |  |  |
|  |  |  | Health Assistant (HA) 5 | |  |  |
|  |  |  | Family welfare assistant (FWA) 6 | |  |  |
|  |  |  | paramedic 7 | |  |  |
|  |  |  | DIC’s workers 8 | |  |  |
|  |  |  | Other Community health workers 9 | |  |  |
|  |  |  | Trained birth attendants (TTBA) 10 | |  |  |
|  |  |  | Traditional birth attendants (TBA) 11 | |  |  |
|  |  |  | BRAC’s UBA 12 | |  |  |
|  |  |  | Hospital’s aya 13 | |  |  |
|  |  |  | Pharmacist 14 | |  |  |
|  |  |  | Kobiraj 15 | |  |  |
|  |  |  | Spiritual healers 16 | |  |  |
|  |  |  | Homeo doctor 17 | |  |  |
|  |  |  | Relatives 18 | |  |  |
|  |  |  | Neighbour 19 | |  |  |
|  |  |  | Others (Specify) 88 | |  |  |
|  |  |  | No one 20 | |  |  |
| 705 | What was your last referral center?  (Single answers acceptable) | | Dhaka Medical College hospital…………………………………..1  Other Government Hospital……………………………………….2  Non-government hospital (Not-for profit) ….……………….3 Name:_______________________________________  Non-government hospital (For profit) ……………….………. 4 Name:_________________________________________  Others (Specify)......................................................8 | | \|  \| \| --- \| |  |
| 706 | What was the distance of last referral centre from your living place? | | _______________km | |  |  |
| 707 | How long did you stay in the health center? | | _____________days  Not applicable=99 | |  |  |
| 708 | Are there any barriers to get referral services? | | Yes…………………………………………………...1  No……………………………………………….…...2 | |  | If no,skip to 801 |
| 709 | If yes, what types of barriers are there? (Multiple answers acceptable) | | Costly/lack of money 1  Not available always /Inconveinient time 2  Do not know where to get 3  Far distance 4  The misbehavior of provider 5  Lack of transport 6  Feeling shameful to get this services 7  Service providers do not like to sell or give services..8  Others (Specify) 88  Don’t know 77 | |  |  |

**Section-8: Respondents perceptions on quality of care with non-medical expectation.**

If you had sought any sexual and reproductive health care from formal health setting/providers in last one year, please, tell us your satisfaction level on quality of care as your expectation

| SI No. | Characteristics | a. Contraceptive use | b. Abortion | c. Maternal Healthcare | d. STI | e. Referral care |
| --- | --- | --- | --- | --- | --- | --- |
| 801 | Sources/places of healthcare in last one year | Public clinic/hospital..........1  Public clinic/hospital (for profit).. ............... ...... ... ...2  NGO clinic/hospital (not for profit)….................................3  Doctor’s Chambers............4  At own home by skilled providers. .. .. .. ......... . .. ..5  Skilled providers’ house …6  Others…………………8 Not applicable………..7 **[**if answer is ‘7’ go to next column**]** | Public clinic/hospital..........1  Public clinic/hospital (for profit).. ............... ...... ... ...2  NGO clinic/hospital (not for profit)….................................3  Doctor’s Chambers............4  At own home by skilled providers. .. .. .. ......... . .. ..5  Skilled providers’ house …6  Others…………………8 Not applicable………..7 **[**if answer is ‘7’ go to next column**]** | Public clinic/hospital..........1  Public clinic/hospital (for profit).. ............... ...... ... ...2  NGO clinic/hospital (not for profit)….................................3  Doctor’s Chambers............4  At own home by skilled providers. .. .. .. ......... . .. ..5  Skilled providers’ house …6  Others…………………8 Not applicable………..7 **[**if answer is ‘7’ go to next column**]** | Public clinic/hospital..........1  Public clinic/hospital (for profit).. ............... ...... ... ...2  NGO clinic/hospital (not for profit)….................................3  Doctor’s Chambers............4  At own home by skilled providers. .. .. .. ......... . .. ..5  Skilled providers’ house …6  Others…………………8 Not applicable………..7 **[**if answer is ‘7’ go to next column**]** | Public clinic/hospital..........1  Public clinic/hospital (for profit).. ............... ...... ... ...2  NGO clinic/hospital (not for profit)….................................3  Doctor’s Chambers............4  At own home by skilled providers. .. .. .. ......... . .. ..5  Skilled providers’ house …6  Others…………………8 Not applicable………..7 **[**if answer is ‘7’ go to next column**]** |
| 802 | Dignity/respect was properly maintained | Not satisfied... .... ... ... ... ...1  A little satisfied...... ... .... ..2  Satisfied... ... ... ... .. ...........3  Moderately satisfied... . ... .4  Highly satisfied... .. ... . .... .5  Silence/no comment/could not remember... ... . ... ... ... 6 | Not satisfied... .... ... ... ... ...1  A little satisfied....... ... ... ..2  Satisfied... ... ... ... .. ...........3  Moderately satisfied... . ... .4  Highly satisfied... .. ... . .... .5  Silence/no comment/could not remember... ... . ... ... ... 6 | Not satisfied... .... ... ... ... ...1  A little satisfied....... ... ... ..2  Satisfied... ... ... ... .. ...........3  Moderately satisfied... . ... .4  Highly satisfied... .. ... . ... .5  Silence/no comment/could not remember... ... . ... ... ... 6 | Not satisfied... .... ... ... ... ...1  A little satisfied....... ... ... ..2  Satisfied... ... ... ... .. ...........3  Moderately satisfied... . ... .4  Highly satisfied... .. ... . ... .5  Silence/no comment/could not remember... ... . ... ... ... 6 | Not satisfied... .... ... ... ... ...1  A little satisfied....... ... ... ..2  Satisfied... ... ... ... .. ...........3  Moderately satisfied... . ... .4  Highly satisfied... .. ... . ... .5  Silence/no comment/could not remember... ... . ... ... ... 6 |
| 803 | Privacy was properly maintained | Not satisfied... .... ... ... ... ...1  A little satisfied...... ... .... ..2  Satisfied... ... ... ... .. ...........3  Moderately satisfied... . ... .4  Highly satisfied... .. ... . .... .5  Silence/no comment/could not remember... ... . ... ... ... 6 | Not satisfied... .... ... ... ... ...1  A little satisfied...... ... .... ..2  Satisfied... ... ... ... .. ...........3  Moderately satisfied... . ... .4  Highly satisfied... .. ... . .... .5  Silence/no comment/could not remember... ... . ... ... ... 6 | Not satisfied... .... ... ... ... ...1  A little satisfied...... ... .... ..2  Satisfied... ... ... ... .. ...........3  Moderately satisfied... . ... .4  Highly satisfied... .. ... . .... .5  Silence/no comment/could not remember... ... . ... ... ... 6 | Not satisfied... .... ... ... ... ...1  A little satisfied...... ... .... ..2  Satisfied... ... ... ... .. ...........3  Moderately satisfied... . ... .4  Highly satisfied... .. ... . .... .5  Silence/no comment/could not remember... ... . ... ... ... 6 | Not satisfied... .... ... ... ... ...1  A little satisfied...... ... .... ..2  Satisfied... ... ... ... .. ...........3  Moderately satisfied... . ... .4  Highly satisfied... .. ... . .... .5  Silence/no comment/could not remember... ... . ... ... ... 6 |
| 804 | Autonomy (patient’s right to get information for choosing treatment options) | Not satisfied... .... ... ... ... ...1  A little satisfied...... ... .... ..2  Satisfied... ... ... ... .. ...........3  Moderately satisfied... . ... .4  Highly satisfied... .. ... . .... .5  Silence/no comment/could not remember... ... . ... ... ... 6 | Not satisfied... .... ... ... ... ...1  A little satisfied...... ... .... ..2  Satisfied... ... ... ... .. ...........3  Moderately satisfied... . ... .4  Highly satisfied... .. ... . .... .5  Silence/no comment/could not remember... ... . ... ... ... 6 | Not satisfied... .... ... ... ... ...1  A little satisfied...... ... .... ..2  Satisfied... ... ... ... .. ...........3  Moderately satisfied... . ... .4  Highly satisfied... .. ... . .... .5  Silence/no comment/could not remember... ... . ... ... ... 6 | Not satisfied... .... ... ... ... ...1  A little satisfied...... ... .... ..2  Satisfied... ... ... ... .. ...........3  Moderately satisfied... . ... .4  Highly satisfied... .. ... . .... .5  Silence/no comment/could not remember... ... . ... ... ... 6 | Not satisfied... .... ... ... ... ...1  A little satisfied...... ... .... ..2  Satisfied... ... ... ... .. ...........3  Moderately satisfied... . ... .4  Highly satisfied... .. ... . .... .5  Silence/no comment/could not remember... ... . ... ... ... 6 |
| 805 | Confidentiality | Not satisfied... .... ... ... ... ...1  A little satisfied...... ... .... ..2  Satisfied... ... ... ... .. ...........3  Moderately satisfied... . ... .4  Highly satisfied... .. ... . .... .5  Silence/no comment/could not remember... ... . ... ... ... 6 | Not satisfied... .... ... ... ... ...1  A little satisfied...... ... .... ..2  Satisfied... ... ... ... .. ...........3  Moderately satisfied... . ... .4  Highly satisfied... .. ... . .... .5  Silence/no comment/could not remember... ... . ... ... ... 6 | Not satisfied... .... ... ... ... ...1  A little satisfied...... ... .... ..2  Satisfied... ... ... ... .. ...........3  Moderately satisfied... . ... .4  Highly satisfied... .. ... . .... .5  Silence/no comment/could not remember... ... . ... ... ... 6 | Not satisfied... .... ... ... ... ...1  A little satisfied...... ... .... ..2  Satisfied... ... ... ... .. ...........3  Moderately satisfied... . ... .4  Highly satisfied... .. ... . .... .5  Silence/no comment/could not remember... ... . ... ... ... 6 | Not satisfied... .... ... ... ... ...1  A little satisfied...... ... .... ..2  Satisfied... ... ... ... .. ...........3  Moderately satisfied... . ... .4  Highly satisfied... .. ... . .... .5  Silence/no comment/could not remember... ... . ... ... ... 6 |
| 806 | Prompt attention | Not satisfied... .... ... ... ... ...1  A little satisfied...... ... .... ..2  Satisfied... ... ... ... .. ...........3  Moderately satisfied... . ... .4  Highly satisfied... .. ... . .... .5  Silence/no comment/could not remember... ... . ... ... ... 6 | Not satisfied... .... ... ... ... ...1  A little satisfied...... ... .... ..2  Satisfied... ... ... ... .. ...........3  Moderately satisfied... . ... .4  Highly satisfied... .. ... . .... .5  Silence/no comment/could not remember... ... . ... ... ... 6 | Not satisfied... .... ... ... ... ...1  A little satisfied...... ... .... ..2  Satisfied... ... ... ... .. ...........3  Moderately satisfied... . ... .4  Highly satisfied... .. ... . .... .5  Silence/no comment/could not remember... ... . ... ... ... 6 | Not satisfied... .... ... ... ... ...1  A little satisfied...... ... .... ..2  Satisfied... ... ... ... .. ...........3  Moderately satisfied... . ... .4  Highly satisfied... .. ... . .... .5  Silence/no comment/could not remember... ... . ... ... ... 6 | Not satisfied... .... ... ... ... ...1  A little satisfied...... ... .... ..2  Satisfied... ... ... ... .. ...........3  Moderately satisfied... . ... .4  Highly satisfied... .. ... . .... .5  Silence/no comment/could not remember... ... . ... ... ... 6 |
| 807 | Access to social support networks during care | Not satisfied... .... ... ... ... ...1  A little satisfied...... ... .... ..2  Satisfied... ... ... ... .. ...........3  Moderately satisfied... . ... .4  Highly satisfied... .. ... . .... .5  Silence/no comment/could not remember... ... . ... ... ... 6 | Not satisfied... .... ... ... ... ...1  A little satisfied...... ... .... ..2  Satisfied... ... ... ... .. ...........3  Moderately satisfied... . ... .4  Highly satisfied... .. ... . .... .5  Silence/no comment/could not remember... ... . ... ... ... 6 | Not satisfied... .... ... ... ... ...1  A little satisfied...... ... .... ..2  Satisfied... ... ... ... .. ...........3  Moderately satisfied... . ... .4  Highly satisfied... .. ... . .... .5  Silence/no comment/could not remember... ... . ... ... ... 6 | Not satisfied... .... ... ... ... ...1  A little satisfied...... ... .... ..2  Satisfied... ... ... ... .. ...........3  Moderately satisfied... . ... .4  Highly satisfied... .. ... . .... .5  Silence/no comment/could not remember... ... . ... ... ... 6 | Not satisfied... .... ... ... ... ...1  A little satisfied...... ... .... ..2  Satisfied... ... ... ... .. ...........3  Moderately satisfied... . ... .4  Highly satisfied... .. ... . .... .5  Silence/no comment/could not remember... ... . ... ... ... 6 |
| 808 | Basic amenities | Not satisfied... .... ... ... ... ...1  A little satisfied...... ... .... ..2  Satisfied... ... ... ... .. ...........3  Moderately satisfied... . ... .4  Highly satisfied... .. ... . .... .5  Silence/no comment/could not remember... ... . ... ... ... 6 | Not satisfied... .... ... ... ... ...1  A little satisfied...... ... .... ..2  Satisfied... ... ... ... .. ...........3  Moderately satisfied... . ... .4  Highly satisfied... .. ... . .... .5  Silence/no comment/could not remember... ... . ... ... ... 6 | Not satisfied... .... ... ... ... ...1  A little satisfied...... ... .... ..2  Satisfied... ... ... ... .. ...........3  Moderately satisfied... . ... .4  Highly satisfied... .. ... . .... .5  Silence/no comment/could not remember... ... . ... ... ... 6 | Not satisfied... .... ... ... ... ...1  A little satisfied...... ... .... ..2  Satisfied... ... ... ... .. ...........3  Moderately satisfied... . ... .4  Highly satisfied... .. ... . .... .5  Silence/no comment/could not remember... ... . ... ... ... 6 | Not satisfied... .... ... ... ... ...1  A little satisfied...... ... .... ..2  Satisfied... ... ... ... .. ...........3  Moderately satisfied... . ... .4  Highly satisfied... .. ... . .... .5  Silence/no comment/could not remember... ... . ... ... ... 6 |
| 809 | Choice of institution/care provider | Not satisfied... .... ... ... ... ...1  A little satisfied...... ... .... ..2  Satisfied... ... ... ... .. ...........3  Moderately satisfied... . ... .4  Highly satisfied... .. ... . .... .5  Silence/no comment/could not remember... ... . ... ... ... 6 | Not satisfied... .... ... ... ... ...1  A little satisfied...... ... .... ..2  Satisfied... ... ... ... .. ...........3  Moderately satisfied... . ... .4  Highly satisfied... .. ... . .... .5  Silence/no comment/could not remember... ... . ... ... ... 6 | Not satisfied... .... ... ... ... ...1  A little satisfied...... ... .... ..2  Satisfied... ... ... ... .. ...........3  Moderately satisfied... . ... .4  Highly satisfied... .. ... . .... .5  Silence/no comment/could not remember... ... . ... ... ... 6 | Not satisfied... .... ... ... ... ...1  A little satisfied...... ... .... ..2  Satisfied... ... ... ... .. ...........3  Moderately satisfied... . ... .4  Highly satisfied... .. ... . .... .5  Silence/no comment/could not remember... ... . ... ... ... 6 | Not satisfied... .... ... ... ... ...1  A little satisfied...... ... .... ..2  Satisfied... ... ... ... .. ...........3  Moderately satisfied... . ... .4  Highly satisfied... .. ... . .... .5  Silence/no comment/could not remember... ... . ... ... ... 6 |

**Section-9:** Observation (Please, write any observations related to respondent’s maternal or reproductive health while interview)

|  |
| --- |

**Section-10: Status of interview**

| SI No. | Ques. | Response | Code | Instruction |
| --- | --- | --- | --- | --- |
| 1001 | Date which data collection started | _____ /______ /2015  day month year |  |  |
| 1002 | Time when data collection started | _____:________  HH MIN |  | railway time |
| 1003 | Time when data collection ended | _____:________  HH MIN |  | railway time |
| 1004 | Place of interview | DIC……………………………….……….……………….……………….………1  Beside Street/spot………………………………….……………………….2  Residence of respondents……………………………………………….3  Other…………………………………..………………………………………….4 |  |  |
| 1005 | Result of the interview | Complete ……………………………………………………………….……...1  Incomplete ………………………………………………..…………………..2 |  | Skip to 1007 if answer ‘1’ |
| 1006 | Reasons of incomplete | Refusal………………………………………………………..…………………1  Absent …………………………………………………..……………………...2  Other…...………………………………………………..………………………3 |  |  |
| 1007 | Data collector’s name -------------------------------------------Date: ------------/--------/2015 | |  |  |
| 1008 | Supervisor’s name -----------------------------------------------Date: ------------/--------/2015 | |  |  |
| 1009 | Data entry person’s name -------------------------------- Date: ------------/--------/2015 | |  |  |

Thank the respondent for their cooperation
